# Defective Zn^2+^ homeostasis in mouse and human platelets with α- and δ-storage pool diseases

**DOI:** 10.1038/s41598-019-44751-w

**Published:** 2019-06-06

**Authors:** Sanjeev Kiran Gotru, Johanna P. van Geffen, Magdolna Nagy, Elmina Mammadova-Bach, Julia Eilenberger, Julia Volz, Georgi Manukjan, Harald Schulze, Leonard Wagner, Stefan Eber, Christian Schambeck, Carsten Deppermann, Sanne Brouns, Paquita Nurden, Andreas Greinacher, Ulrich Sachs, Bernhard Nieswandt, Heike M. Hermanns, Johan W. M. Heemskerk, Attila Braun

**Affiliations:** 1 Institute of Experimental Biomedicine, University Hospital and Rudolf Virchow Center, University of Würzburg, Würzburg, Germany; 2 Department of Biochemistry, CARIM, Maastricht University, Maastricht, The Netherlands; 3 Practice for Pediatric Hematology and Hemostaseology, Munich, Germany; 4 Haemostasikum, Munich, Germany; 5 Institut Hospitalo-Universitaire LIRYC, Plateforme Technologique d’Innovation Biomédicale, Hôpital Xavier Arnozan, Pessac, France; 6 Institute for Immunology and Transfusion Medicine, University Medicine Greifswald, Greifswald, Germany; 7 Institute for Clinical Immunology and Transfusion Medicine, Justus Liebig University, Giessen, Germany; 8 Medical Clinic and Policlinic II, Division of Hepatology, University Hospital Würzburg, Würzburg, Germany

**Keywords:** Coagulation system, Metals, Coagulation system, Metals

## Abstract

Zinc (Zn^2+^) can modulate platelet and coagulation activation pathways, including fibrin formation. Here, we studied the (patho)physiological consequences of abnormal platelet Zn^2+^ storage and release. To visualize Zn^2+^ storage in human and mouse platelets, the Zn^2+^ specific fluorescent dye FluoZin3 was used. In resting platelets, the dye transiently accumulated into distinct cytosolic puncta, which were lost upon platelet activation. Platelets isolated from *Unc13d*^−/−^ mice, characterized by combined defects of α/δ granular release, showed a markedly impaired Zn^2+^ release upon activation. Platelets from *Nbeal2*^−/−^ mice mimicking Gray platelet syndrome (GPS), characterized by primarily loss of the α-granule content, had strongly reduced Zn^2+^ levels, which was also confirmed in primary megakaryocytes. In human platelets isolated from patients with GPS, Hermansky-Pudlak Syndrome (HPS) and Storage Pool Disease (SPD) altered Zn^2+^ homeostasis was detected. In turbidity and flow based assays, platelet-dependent fibrin formation was impaired in both *Nbeal2*^−/−^ and *Unc13d*^−/−^ mice, and the impairment could be partially restored by extracellular Zn^2+^. Altogether, we conclude that the release of ionic Zn^2+^ store from secretory granules upon platelet activation contributes to the procoagulant role of Zn^2+^ in platelet-dependent fibrin formation.

## Introduction

Zinc (Zn^2+^) is an essential micronutrient, which modulates several enzymes, regulates the structure of zinc finger domains, induces diverse signaling pathways as a second messenger, and acts as an important cofactor in the metabolism^1^. Zn^2+^ circulates in the blood plasma at a concentration of 10–20 µM. However, only small amounts (0.1–2 µM) are present in the free ionic form, which can be taken up by platelets and other circulating blood cells^2,3^. Reduced Zn^2+^ uptake in the body results in altered platelet aggregation responses and impaired hemostasis, while intracellular chelation of Zn^2+^ in platelets inhibits tyrosine phosphorylation cascades as well as platelet reactivity and aggregation responses^4–6^. Interestingly, the Zn^2+^ concentration is considerably higher in platelets than in blood plasma^7^. Earlier findings showed that incubation with extracellular Zn^2+^ increases the Zn^2+^ concentration in the platelet cytoplasm and granules, pointing to the existence of Zn^2+^ uptake and storage mechanisms in these cells. Furthermore, the Zn^2+^ concentration in blood serum was found to be higher than in plasma, suggesting that activated platelets can release a significant amount of stored Zn^2+^ during clotting^7^. It has been suggested by some reports that protein-bound Zn^2+^ is accumulated in the platelet α-granules, which was explained by its high affinity for fibrinogen, albumin, histidine-rich glycoprotein, and factor XIII^8^. Extracellular Zn^2+^ directly binds fibrinogen and changes the fibrin fiber diameter, which is accompanied by increased clot stability^9,10^, raising the possibility that extracellular Zn^2+^ in the blood plasma, *e*.*g*. released from platelets, supports fibrin clot formation.

α-, δ- Storage pool disease (SPD) is characterized by deficiency of either α- or δ-granules or both types of granules in platelets. The Gray Platelet Syndrome in mouse^11,12^ and human^13–15^ is associated with an abolished gene function of *Nbeal2 (NBEAL2)*, where primarily α-granule formation is severely impaired^14,15^. Mice lacking the *Unc13d* gene have an abolished δ-granule secretion and also partially defective exocytosis of α-granules and lysosomes^16^. These granular defects result in platelet dysfunction and severely prolonged tail-bleeding times in both, *Unc13d*^−/−^ and *Nbeal2*^−/−^ mice^17^.

In the present study, we show that the free ionic form of Zn^2+^ has a granular localization in both human and murine platelets, which is rapidly lost upon platelet activation. Characterization of *Nbeal2*^−/−^ and *Unc13d*^−/−^ strongly suggests that free zinc is mainly stored in α-granules. Further results highlight the importance of the platelet Zn^2+^ store and release in the modulation of coagulation and fibrin formation.

## Results and Discussion

**Figure 1:**
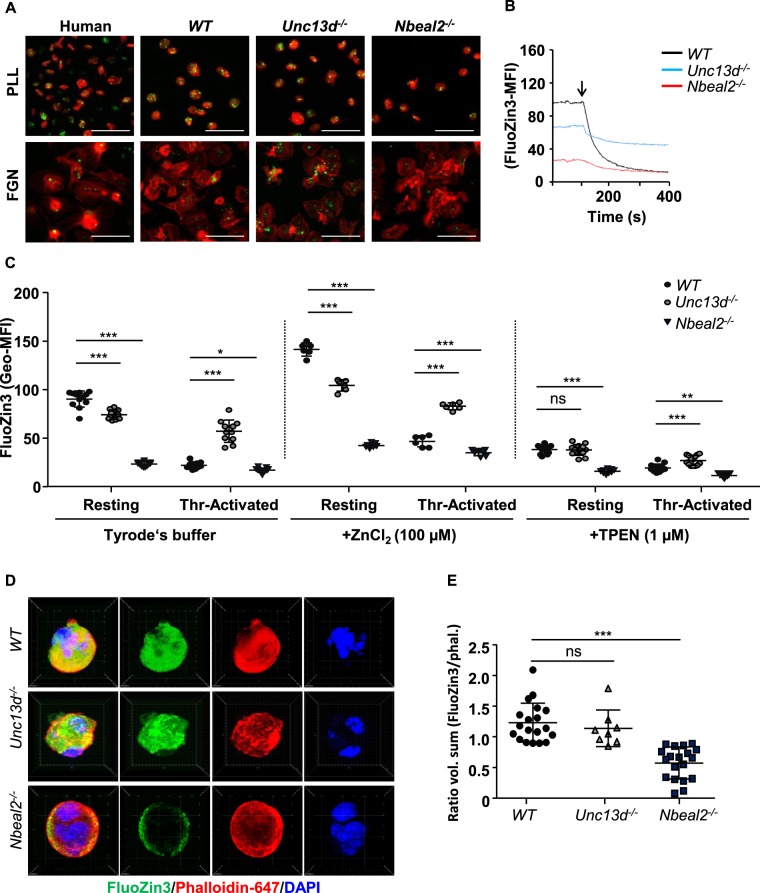
Distribution and levels of Zn^2+^ in human and mouse platelets. (**A**) Human (control) and mouse platelets loaded with FluoZin3 (green) were fixed (upper panels) or allowed to adhere on poly-lysine L (PLL) or spread on fibrinogen (FGN), (lower panels), stained with Atto 647N-phalloidin (red), and examined by confocal microscopy (representative images, Scale bar: 5 µm). (**B**) Washed *WT*, *Unc13d*^−/−^ and *Nbeal2*^−/−^ mouse platelets were loaded with FluoZin3, stimulated with thrombin (Thr-Activated, 0.1 U/mL), and fluorescence changes were observed by flow cytometry (MFI: mean fluorescence intensities). (**C**) Quantification of [Zn^2+^]_i_ in mouse platelets, both resting and after thrombin stimulation (Thr-Activated); pre-incubation of *WT*, *Unc13d*^−/−^ and *Nbeal2*^−/−^ platelets with 100 μM ZnCl_2_ or 1 μM TPEN. Complete kinetic curve was recorded upon appending the first 50 sec (initial stage: Resting) to 400 sec (end point: Thr-Activated). Average of initial and end point measurements was shown. Each dot represents an individual mouse, Mean ± SEM. (**D**) *In vitro* differentiated bone-marrow megakaryocytes (MKs) from *WT*, *Unc13d*^−/−^ and *Nbeal2*^−/−^ mice were loaded with FluoZin3 (green), and then fixed (left panels) or allowed to adhere on poly-lysine L (PLL). Cells were examined by confocal microscopy (representative images); staining Atto 647N-phalloidin (red) and DAPI (blue). Each dote represents an independent experiment, n = 4 mice per group, Mean ± SEM. (**E**) Quantification of [Zn^2+^]_i_ in FluoZin3-loaded MKs. Ratiometric analyses between FluoZin3 and phalloidin was shown. *P < 0.05; **P < 0.01; ***P < 0.001. 2-way ANOVA, Bonferroni’s multiple comparisons test, Student’s t-test.

Given the unclearness of the location of intra-platelet ionic Zn^2+^ and the role of platelet Zn^2+^ release in clot formation, we re-evaluated these topics in human and mouse platelets. Loading of control human and *WT* mouse platelets with the Zn^2+^ specific fluorescence dye, FluoZin3, indicated that the cells of either species at resting state contained several stained foci, which became markedly reduced upon platelet spreading on a fibrinogen-coated surface. Some of the activated platelets became completely negative for FluoZin3 staining (Fig. 1A). This suggested that a substantial part of the intracellular free Zn^2+^ concentration ([Zn^2+^]_i_) in platelets is concentrated in granules, which are released upon activation. This was confirmed for FluoZin3-stained platelets in suspension measurement of the fluorescence intensity over time using flow cytometry (Fig. 1B). Addition of thrombin to the mouse platelet suspension caused a rapid and strong decrease in [Zn^2+^]_i_, (approximately 60–70%), (Fig. 1B). Taken together, this pointed to platelet secretory granules as major Zn^2+^ stores, although a remaining part of the Zn^2+^ can be available for Zn^2+^-binding proteins, such as metallothionein isoforms, as previously detected in megakaryocytes (MKs) and platelets^18,19^.

Release of the α- and δ-granule content from activated platelets affects several processes in the blood, including coagulation, wound repair and inflammation. Platelets from *Unc13d*^−/−^ mice are characterized by an abolished δ-granule release and reduced α-granule secretion^16^. To study whether this defect in granule release is accompanied by a defect in Zn^2+^ efflux, we loaded platelets from both wild-type (*WT*) and *Unc13d*^−/−^ mice with FluoZin3. In comparison to *WT*, platelets from *Unc13d*^−/−^ mice showed a slightly reduced basal [Zn^2+^]_i_, but a severely impaired Zn^2+^ efflux after thrombin stimulation (Fig. 1A–C). Similarly, we used *Nbeal2*^−/−^ mice, as a model of Gray Platelet Syndrome (GPS) lacking the α-granule content, which -in contrast to *Unc13d*^−/−^ mice showed already under resting conditions severely reduced intracellular Zn^2+^ level, which upon activation were not much more reduced (Fig. 1A–C; Supplementary Fig. 1). To investigate further whether abnormal granular Zn^2+^ store of MKs could account for the dysregulated Zn^2+^ homeostasis in *Nbeal2*^−/−^ platelets, bone marrow cells were differentiated to MKs *in vitro*. Subsequently, the FluoZin3 stained MKs were analyzed by confocal microscopy (Fig. 1D). *Unc13d*^−/−^ MKs showed similar, homogenous FluoZin3 staining as *WT* MKs. However, abnormal accumulation of FluoZin3 staining close to the plasma membrane was observed in *Nbeal2*^−/−^ MKs with strongly reduced fluorescence intensity within the cytoplasm (Fig. 1D,E). This suggested that an altered granular Zn^2+^ content in *Nbeal2*^−/−^ MKs is a primary cause of the reduced [Zn^2+^]_i_ in mutant platelets. To correlate our results to human platelet disorders, we isolated platelets from GPS^13^, HPS (Hermansky-Pudlak Syndrome), (Supplementary Table 1, Supplementary Fig. 2C) and SPD (Storage Pool Disease), (Supplementary Table 2) patients. Indeed, strongly reduced Zn^2+^ content was observed in resting and activated platelets from the GPS patient (Supplementary Fig. 2A) which resembles the observation made in *Nbeal2*^−/−^ mice. Platelets isolated from the HPS patient had a reduced intracellular concentration of ionic Zn^2+^  under resting conditions and released lower amounts of Zn^2+^ compared to platelets from a healthy donor upon activation with thrombin. Similar observations were made in a number of patients with SPD (Supplementary Fig. 2B). Our preliminary results therefore suggest that Zn^2+^ homeostasis seems to be impaired in human platelets with granular abnormalities. Further investigations are necessary to confirm these results in a large cohort of patients.

To investigate further whether defective Zn^2+^ influx may contribute to the reduced [Zn^2+^]_i_ in mutant platelets, we incubated *WT*, *Unc13d*^−/−^ and *Nbeal2*^−/−^ platelets with either ZnCl_2_ or the Zn^2+^ chelator TPEN as a negative control. We then measured [Zn^2+^]_i_ concentrations again with FluoZin3. Even though the addition of ZnCl_2_ increased the basal [Zn^2+^]_i_ in all mutant platelets, this level still did not reach that in *WT* platelets (Fig. 1C). This result is compatible with a regular Zn^2+^ uptake, but a defective storage in the mutant platelets. Markedly, TPEN treatment reduced the Zn^2+^ level in a similar manner in *WT* and *Unc13d*^−/−^ platelets, but not in *Nbeal2*^−/−^ platelets (Fig. 1C); suggesting that the free ionic Zn^2+^ store in *Nbeal2*^−/−^ platelets is limited. Altogether, these alterations may point to an impaired Zn^2+^ transport into the (low numbers of) available secretory granules. In mammalians, several protein members of the ZIP protein family can mediate Zn^2+^ influx, thereby increasing [Zn^2+^]_i_ concentrations. On the other hand, isoforms of the ZnT family regulate Zn^2+^ efflux from the cytosol to the extracellular space or into intracellular organelles lowering [Zn^2+^]_i_ concentration^1^. Using *in vitro* grown primary MKs and quantitative RT-PCR, we confirmed mRNA expression of several ZIP/ZnT family members in this cell type (Supplementary Fig. 3). Whether the expression profiles of ZIP/ZnT transporters are altered in *Nbeal2*^−/−^ and *Unc13d*^−/−^ platelets, still needs to be investigated at mRNA and protein levels.

**Figure 2:**
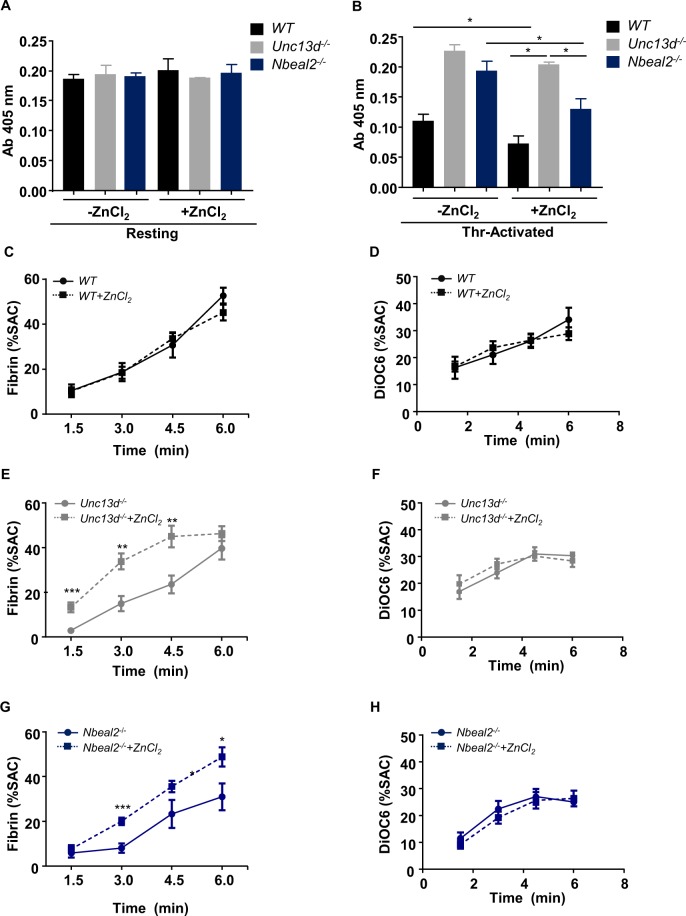
Effect of Zn^2+^ on fibrin formation under static and flow conditions in mouse models with defective α- and δ-granule biogenesis or secretion. (**A**,**B**) Turbidity assay on (**A**) resting and (**B**) thrombin-activated platelets. (**B**) *WT*, *Unc13d*^−/−^ and *Nbeal2*^−/−^ platelets were activated with 0.1 U/mL thrombin in the presence or absence of 100 μM ZnCl_2_, and turbidity was measured at 405 nm in an ELISA reader, n = 3 mice per group, Mean ± SEM. (**C–H**) Citrated whole-blood from *WT*, *Unc13d*^−/−^ or *Nbeal2*^−/−^ mice, labelled with DiOC_6_ (0.5 μg/mL) and AF647-fibrin(ogen), (8.5 μg/mL) in the presence or absence of 100 μM ZnCl_2_, was flowed under recalcification over a collagen surface for 8 min at a shear rate of 1,000 s^−1^. Platelet adhesion and fibrin formation were assessed by brightfield and multicolor fluorescence microscopy in time. Times to first fibrin formation recorded for individual flow runs. (**C**–**H**) Quantification of (**C**,**E**,**G**) fibrin and (**D**,**F**,**H**) adherent platelets. n = 6 mice per group, Mean ± SEM. SAC: surface adherent coverage. *P < 0.05; **P < 0.01; ***P < 0.001. 2-way ANOVA, Bonferroni’s multiple comparisons test, Student’s t-test.

It is known that platelet-released Zn^2+^ can modulate local coagulant reactions, including contact activation and fibrin clotting^9,10,20^. Therefore, we considered that a defective granule biogenesis or granule secretion altering platelet Zn^2+^ release, may also affect the fibrin clotting process. First, a turbidity assay was performed to quantify the thrombin-induced fibrin formation according to the changes in the absorbance at 405 nm. *WT*, *Nbeal2*^−/−^ and *Unc13d*^−/−^ platelets were incubated in the presence or absence of extracellular Zn^2+^, prior to the activation with thrombin (Fig. 2A,B). We found that turbidity was lower in *WT* platelet-releasate, which was further decreased in the presence of extracellular Zn^2+^ (Fig. 2B). In *Nbela2*^−/−^ mice turbidity was similar to resting level in platelet releasate after thrombin activation (Fig. 2A), but it was significantly reduced in the presence of Zn^2+^ (Fig. 2B). However, extracellular Zn^2+^ cannot fully restore fibrin formation to the *WT* level, due to the strongly reduced fibrinogen content and release from α-granules of *Nbela2*^−/−^ platelets. No significant change was found in platelet releasate from *Unc13d*^−/−^ mice neither in the presence nor in the absence of Zn^2+^, likely due to the abolished δ-granule and reduced α-granule secretion which strongly reduced fibrin clot formation in this experimental condition (Fig. 2B). To visualize fibrin formation, releasate of *WT* and mutant platelets were objected to scanning electron microscopy (SEM), and ultrastructure of fibrin clot was obviously different between *WT*, *Unc13d*^−/−^ and *Nbela2*^−/−^ platelets and the structure of fibrin clot was modified in the presence of Zn^2+^ (Supplementary Fig. 4A). However, further investigation is necessary to understand the exact role of platelet Zn^2+^ release in this process. Previous studies showed that 70% of α granules cannot be released in *Unc13d*^−/−^ platelets due to the lack of ADP-mediated amplification of α-granule secretion^16^. To investigate ADP -dependent fibrin formation, turbidity assays were performed differently, in the absence (Supplementary Fig. 4B) or the presence (Supplementary Fig. 4C) of high dose of apyrase, stimulating platelet granule release with thrombin (1 U/mL). Similar results were obtained in these experimental settings as before, suggesting that Zn^2+^-induced fibrin formation does not require ADP at high dose of thrombin.

Recording of the fibrin formation on platelet thrombi during whole-blood flow over collagen/tissue factor microspots provides an adequate way to evaluate hemostatic activity *ex vivo*^21^. Using microfluidics and a wall-shear rate of 1000 s^−1^, we assessed the kinetics of fibrin clot formation at such microspots from the accumulation rate of fluorescently-labeled platelets and fibrin (Supplementary Fig. 5). In blood samples from *WT* mice, Zn^2+^ addition did not change the kinetics of fibrin formation, thus indicating that Zn^2+^ was not a limiting factor in this process (Fig. 2C,D). Similar flow experiments were performed with blood from mice carrying the platelet secretion defects. Markedly, the platelet-dependent fibrin clot formation under flow was impaired in whole blood samples from *Unc13d*^−/−^ (Fig. 2E,F) and *Nbeal2*^−/−^ mice (Fig. 2G,H), but in both cases the kinetics of fibrin formation was accelerated by Zn^2+^ supplementation.

Altogether, our data significantly extend the early observation in 1985^1^, that extracellular Zn^2+^ as well as platelet Zn^2+^ release have a procoagulant effect. In addition, our data indicate that several genetic dysorders impairing platelet granular content or release, especially in patients with GPS, could negatively influence platelet Zn^2+^-dependent fibrin formation and this defect could be partially rescued by Zn^2+^ supplementation. Our results also suggest that determination of the platelet Zn^2+^ content with FluoZin3 could be a novel prognostic biomarker for patients related storage pool disease and other bleeding disorders.

## Material and Methods

### Reagents

FluoZin3/AM (F24195, Invitrogen), N,N,N′,N′-tetrakis 2-pyridylmethyl ethylene diamine (TPEN) (ALX-450-011-M100, Enzo). Horm collagen (11207690, Nycomed), tissue factor (TF) was from Innovin (B4212-40, Dade-Behring, Marburg, Germany). Membrane dye DiOC_6_ was from AnaSpec. AF568-annexin A5 was from Life Technology (A13202, Eugene, USA) and AF647-labeled fibrinogen from Molecular Probes (F35200, Life Technology). Human fibrinogen (F4883) was from Sigma-Aldrich and thrombin from (10602400001) was from Roche Diagnostics. Phalloidin-AF647 (65906) was from Sigma-Aldrich. NucleoSpin®RNA II Kit was from Macherey-Nagel. High Capacity cDNA RT Kit was from Applied Biosystems and FastStart Universal SYBR Green Master Mix was from Roche. All reagents were bought from German suppliers unless otherwise stated.

### Healthy subjects, patients and blood taking

All patients and blood donors were volunteers who gave free and informed written consent to participate in this study, conforming to the ethical standards adhering to the local Institutional Review Boards and the Declaration of Helsinki. Ethics committee of University of Würzburg Germany (reference: 52/15), and INSERM, France (reference: RBM-04-14) approved the study. Peripheral blood counts were within the reference ranges. SPD patients were diagnosed by standard routine diagnostics in the clinical centers showing the reported ADP/ATP content and release, as shown in Supplementary Table 2. The Gray Platelet Syndrome (GPS) patient was published earlier^13^ and Hermansky-Pudlak syndrome patient characterized by standard routine diagnostics and Transmission Electron Microscopy (TEM) in the respective clinical center, as shown in Supplementary Fig. 2C and Supplementary Table 1.

### Animals

*Unc13d*^−/−^ or *Nbeal2*^−/−^ knockout mice have been previously described^11,17^. All experiments with animals were performed in accordance with the German legislation and guidelines of University of Würzburg and Regional Administration of Unterfranken (Lower District), Würzburg, Germany.

### Confocal microscopy

For confocal microscopy, platelets (5 × 10^5^/µL) were loaded with FluoZin-3/AM (5 µg/mL of dye in DMSO mixed 1:1 in 37 °C pre-heated pluronic acid), left shaking at 300 rpm; 30 min; 37 °C; dark. To allow the ester to cleave, loaded platelets are left idle for 20 min; 37 °C; dark. 125,000 of FluoZin3-AM loaded platelets/µL were fixed with 2% PFA-Phem, and allowed to adhere immediately on poly-L-lysine (for 20 min at room temperature), or allowed to spread on cover slips coated with fibrinogen (100 µg/mL) at 37 °C for 30 min after 0.01 U/mL thrombin stimulation (0.2 mM CaCl_2_ supplemented). The spread platelets were fixed with 2% PFA-Phem for 20 min. Adhered platelets were counter-stained with 100 µL of phalloidin-Atto 647 N (diluted 1:200 in PBS) for 1 h. Coverslips were washed with PBS, and mounted onto glass slides using Fluro shield mounting medium. Microscopic images were obtained using a Leica TCS SP5 confocal microscope (Leica Microsystems, Wetzlar, Germany), and analyzed with FiJi, Leica light and Imaris software.

### Turbidity assay for fibrin formation

Washed platelets from *WT*, *Unc13d*^−/−^ and *Nbeal2*^−/−^ mice were activated with 0.1 U/mL or 1 U/mL thrombin in the presence or absence of 100 μM ZnCl_2_ or apyrase (2 U/mL) and were then incubated at 37 °C for 30 min. Thrombin-induced fibrin polymerisation was monitored for 2 hours by evaluating the turbidity at 405 nm using an ELISA reader.

### Whole-blood fibrin formation on platelet thrombi in a flow system

Platelet-mediated fibrin formation in recalcified mouse whole blood using a microfluidics flow chamber with microspots of collagen and tissue factor, at a shear rate of 1000 s^−1^ was measured, as described before^22^.

### Statistical analysis

Statistical significance was analyzed using SigmaPlot 11 software. Statistical difference of the means (Mean ± SEM) was determined using 2-way analysis of variance, followed by the stated test of variance for significance or an unpaired Student’s t test. *P* values < 0.05 were considered to be significant (*P < 0.05, **P < 0.01, ***P < 0.001, ****P < 0.0001).

## Supplementary information

Supplementary information
