# Supplementary material for: Defective Zn2+ homeostasis in mouse and human platelets with α- and δ-storage pool diseases
Source: Sci Rep. 2019 Jun 6;9:8333. doi: 10.1038/s41598-019-44751-w (PMC6554314; doi:10.1038/s41598-019-44751-w)
Supplement: Supplementary file 1 — Supplementary information [file 41598_2019_44751_MOESM1_ESM.pdf]

Defective Zn^{2+} homeostasis in mouse and human platelets with α- and δ-storage pool diseases

Sanjeev Kiran Gotru[1,*], Johanna P. van Geffen[2,*], Magdolna Nagy[2], Elmina Mammadova-Bach[1], Julia Eilenberger[3], Julia Volz[1], Georgi Manukjan[1], Harald Schulze[1], Leonard Wagner[8], Stefan Eber[3], Christian Schambeck[4], Carsten Deppermann[1], Sanne Brouns[2], Paquita Nurden[5], Andreas Greinacher[6], Ulrich Sachs[7], Bernhard Nieswandt[1], Heike M. Hermanns[8], Johan W. M. Heemskerk[2], and Attila Braun[1]

[1]Institute of Experimental Biomedicine, University Hospital and Rudolf Virchow Centre, University of Würzburg, Germany; [2]Department of Biochemistry, CARIM, Maastricht University, The Netherlands; [3]Practice for Pediatric Hematology and Hemostaseology, Munich, Germany; [4]Haemostasikum, Munich, Germany, [5]Institut Hospitalo-Universitaire LIRYC, Plateforme Technologique d'Innovation Biomédicale, Hôpital Xavier Arnozan, Pessac, France; [6]Institute for Immunology and Transfusion Medicine, University Medicine Greifswald, Germany; [7]Institute for Clinical Immunology and Transfusion Medicine, Justus Liebig University Giessen, Germany; [8]Medical Clinic and Policlinic II, Division of Hepatology, University Hospital Würzburg, Würzburg, Germany

Running Title: Defective platelet Zn^{2+} homeostasis

* These authors contributed equally

Corresponding author: Attila Braun, University Hospital of Würzburg and Rudolf Virchow Centre, Josef-Schneiderstr. 2 D15, 97080 Würzburg, Germany, e-mail: attila.braun@virchow.uni-wuerzburg.de

Supplementary information is available online:

Supplementary Materials and Methods:

Platelet preparation and washing

Mice were bled under isofluran anesthesia from the retroorbital plexus. 700 µL blood were collected in a reaction tube containing 300 µL heparin in Tris-buffered saline (20 U/ml, pH 7.3) or 300 µL Acid-citrate-dextrose (ACD, 85 mM trisodium citrate dehydrate, 71 mM citric acid monohydrate, 111 mM D-glucose, pH 4.5). Blood was centrifuged at 800 rpm for 6 min at RT. Supernatant and buffy coat were transferred into a new tube to obtain platelet rich plasma (PRP). To prepare washed platelets, PRP was centrifuged at 2,800 rpm for 5 min at RT and the pellet was resuspended in 1 mL Ca^{2+}-free Tyrode's buffer containing apyrase (0.02 U/mL) and prostaglandin (PGI_2, 0.5 µM). After 10 min incubation at 37°C, the sample was centrifuged at 2,800 rpm for 5 min. After a second washing step, the platelet pellet was resuspended in the appropriate volume of Tyrode's buffer containing apyrase (0.02 U/mL) and left to incubate for at least 30 min at 37°C before analysis.

Flow cytometry

Washed platelets ($5x10^5$ cells/µL) were loaded with FluoZin-3/AM as described above. 25 µL of the loaded platelets were diluted to 1 mL with Tyrodes' Buffer without $CaCl_2$, and fluorescence intensity was recorded for 50 sec. Subsequently, 500 µL of the platelet suspension was added to 0.2 mM $CaCl_2$, 0.01 U/mL thrombin and fluorescence events were recorded over time by flow cytometry (until 400 sec). Measurement was shown as complete kinetic curve upon appending the first 50 sec to the rest of the measurement. Platelets were pre-incubated with either 1 µM of Zn^{2+} chelator TPEN or 100 µM of $ZnCl_2$ as indicated. Flow cytometric measurements were performed on BD FACS Canto II and results analyzed using FlowJo software. Approximately, initial and final 10 % of each dot plot was considered. FITC Geo-mean fluorescent intensity was presented with each symbol representing a single

measurement from a single mouse or individual as is the case.

In vitro bone marrow megakaryocyte isolation and differentiation

Bone marrow cells were obtained from femur and tibia by flushing and then were cultured in 2.6% nutrient-supplemented StemPro medium with 2 mM L-glutamine, 100 IU mL^{-1} penicillin, 50 mg/mL^{-1} streptomycin and 20 ng/mL^{-1} murine stem cell factor and 50 ng/mL^{-1} of murine thrombopoietin at 37°C under 5% CO_2 for 3 days. Mature megakaryocytes were enriched by a one-step BSA gradient, as previously described [1].

RNA isolation and Quantitative Real-Time Polymerase Chain Reaction (qRT-PCR)

Total RNA from matured megakaryocytes at day 5 was extracted using the NucleoSpin®RNA II Kit according to manufacturer's instructions. RNA was reverse transcribed using the High Capacity cDNA RT Kit. qRT-PCR was performed using the FastStart Universal SYBR Green Master Mix. Relative values of gene expression were normalized to Ribosomal Protein Large Subunit P0 (Rplp0). Rplp0 expression was set to 100 and values were presented in logarithmic scale. Primer sequences are presented in Supplementary Table 3.

Scanning Electron Microscopy (SEM)

Thrombin-activated platelet releasates were fixed on coverslips overnight with 2.5% glutaraldehyde in 0.1 M sodium cacodylate buffer, then washed three times in 0.9% saline and dehydrated sequentially in increasing concentrations of ethanol. Samples were air-dried with hexamethyldisilazan, sputtered with gold, and examined under Phenom Scanning Desktop Electron Microscope.

Transmission Electron Microscopy (TEM)

Platelet rich plasma (PRP) was fixed with 2.5% glutaraldehyde in 50 mM cacodylate buffer. Ultrathin sections of the fixed samples were stained in 2% uranyl acetate after embedding in epon 812. Later samples were stained with 2% uranyl acetate and lead citrate. Images were acquired using EM900 transmission electron microscope (Carl Zeiss, Germany).

Supplementary Figure Legends

Supplementary Figure 1. Quantification of FluoZin3 content in platelets. Quantification of the percentage region of interest (% ROI) calculated from green channel of the Z-stacks (FluoZin3) expressed in arithmetic units (AU) in (**A**) resting and (**B**) thrombin-activated *WT*, *Unc13d*$^{-/-}$ and *Nbeal2*$^{-/-}$ mouse platelets. n=3 mice per group, Mean±SEM. Images were obtained using Leica TCS SP5 confocal microscope (Leica Microsystems, Wetzlar, Germany) and analyzed with using Fiji, Leica LAS AF Lite and Imaris softwares.

Supplementary Figure 2. Dysregulated Zn^{2+} homeostasis in human platelets with defective α- and δ-granule biogenesis or secretion. (**A**) Washed human platelets isolated from healthy subject (control: Ctrl), Hermansky-Pudlak Syndrome (HPS) and Gray Platelet Syndrome (GPS) patients were loaded with FluoZin3, stimulated with thrombin (0.01 U/mL) and fluorescence changes (MFI: mean fluorescence intensities) were observed by flow cytometry. Representative kinetic curves of Ctrl (black line), HPS (blue line) and GPS (red line) human platelets are shown. Arrows indicate agonist addition. (**B**) Relative Zn^{2+} content and release in human SPD platelets using FluoZin3, both resting and after thrombin stimulation (Thr-Activated). (**C**) Representative transmission electron microscopy (TEM) images of a healthy subject (Ctrl) and a HPS patient. Scale bar: 2µm. ***P*<0.01; ****P*<0.001. Student's t-test**.**

Supplementary Figure 3. Relative mRNA expression of Znt and Zip family members in mature megakaryocytes. Expression of Znt /Zip is represented in logarithmic scale. n=3 mice, Mean ±SEM.

Supplementary Figure 4. Effect of Zn^{2+} on fibrin formation under static conditions in mouse models with defective α- and δ-granule biogenesis or secretion. (A) Representative scanning electron microscopy images of releasate of *WT*, *Unc13d*$^{-/-}$ and

Nbeal2$^{-/-}$ platelets. Ultrastructure of fibrin clots are shown**.** (**B**, **C**) Turbidity assay on thrombin-activated platelets. (**B**) *WT*, *Unc13d*$^{-/-}$ and *Nbeal2*$^{-/-}$ platelets were activated with 1U/mL thrombin in the (**B**) absence or (**C**) presence of Apyrase (2U/mL) and 100 μM $ZnCl_2$ and turbidity was measured at 405 nm in an ELISA reader. n=4 mice, Mean ±SEM.**P*<0.05; 2-way ANOVA, Bonferroni's multiple comparisons test.

Supplementary Figure 5. Whole-blood fibrin formation on platelet thrombi in a flow system. Representative brightfield and fluorescence microscopic images of thrombi from mouse platelets (green) and fibrin fibers extending from the thrombi (red) after 6 min.

Supplementary Table 1. Hermansky-Pudlak syndrome (HPS) patient platelets were characterized as presented. Values in grey box are in pathological range. Platelets from HPS patient were analyzed using standard analysis techniques for bleeding score, platelet count and size as indicated above and values reported. Age, sex, bleeding score, platelet count and size, platelet aggregation, ATP release and ADP content of HPS patient platelets were presented. Platelet function test using PFA-100 Analyzer was performed in HPS patient platelets upon stimulation with indicated agonists. STHG-BAT score: International Society on Thrombosis and Haemostasis Bleeding Assessment Tools, MPV: mean platelet volume, fL: femtolitre, PFA: Platelet Function Assays, Col: Collagen, ADP: Adenosine diphosphate, ATP: Adenosine triphosphate. Agg: Aggregation, TRAP: Thrombin receptor-activating peptide.

Supplementary Table 2. Storage pool disease (SPD) patient platelets were characterized as presented. Values in grey box are in pathological range. Platelets from SPD patients were analyzed using standard analysis techniques for bleeding score, platelet count and size as indicated above and values reported. Age, sex, bleeding score, platelet count and size, platelet aggregation, ATP release and ADP content of SPD patient platelets were presented. Platelet function test using PFA-100 Analyzer was performed in SPD patient platelets upon stimulation with indicated agonists. STHG-BAT score: International Society on Thrombosis and Haemostasis Bleeding Assessment Tools, MPV: mean platelet volume, fL: femtolitre, PFA:

Platelet Function Assays, Col: Collagen, ADP: Adenosine diphosphate, ATP: Adenosine triphosphate. Agg: Aggregation, TRAP: Thrombin receptor-activating peptide.

Supplementary Table 3. List of primer sequence

References

1 Nakeff, A. & Floeh, D. P. Separation of megakaryocytes from mouse bone marrow by density gradient centrifugation. *Blood* **48**, 133-138 (1976).

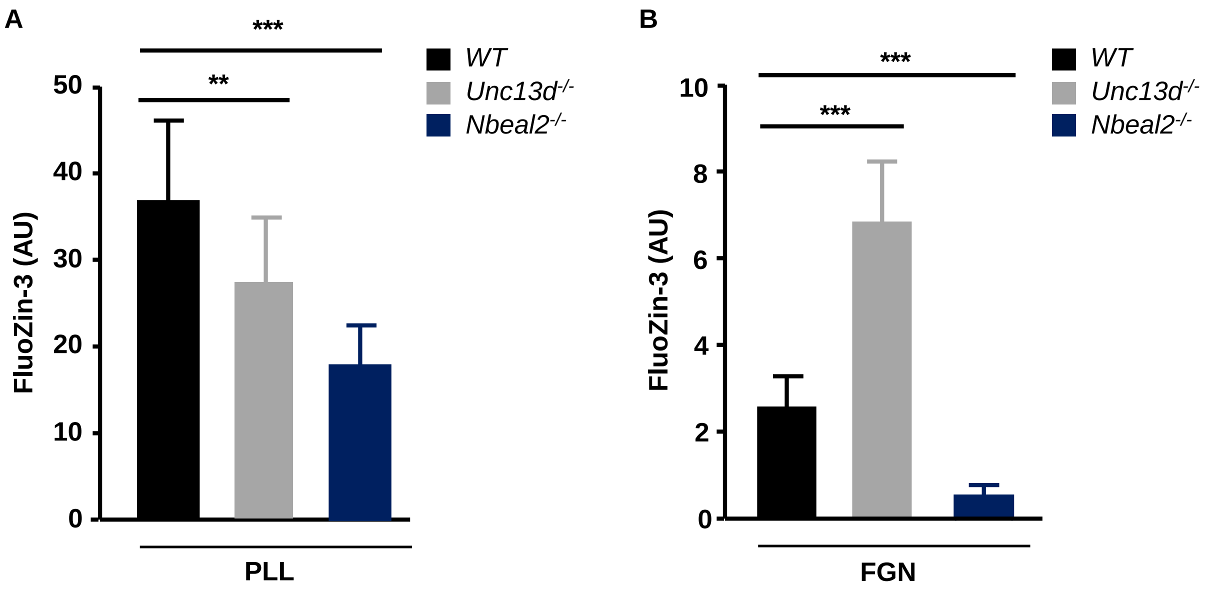

Supplementary Figure 1

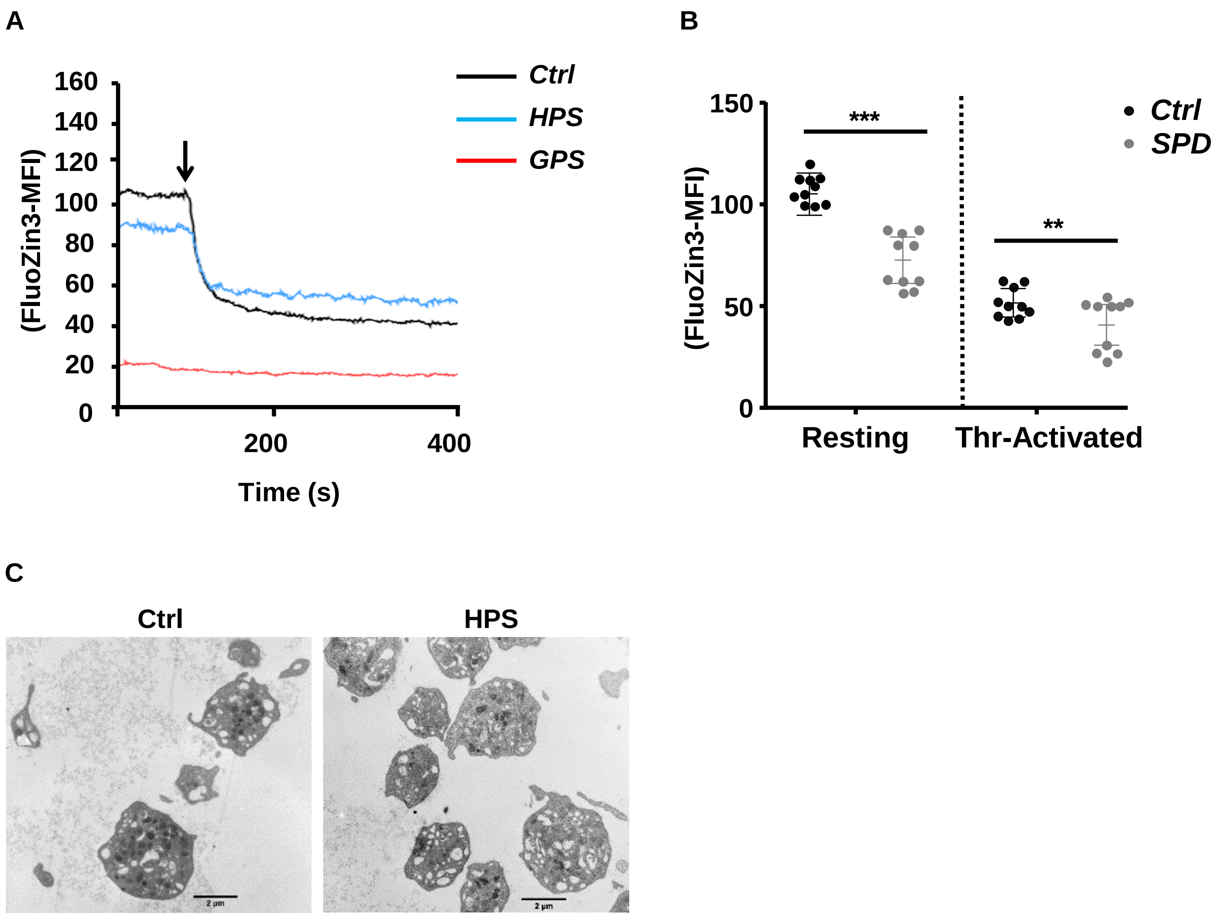

Supplementary Figure 2

A

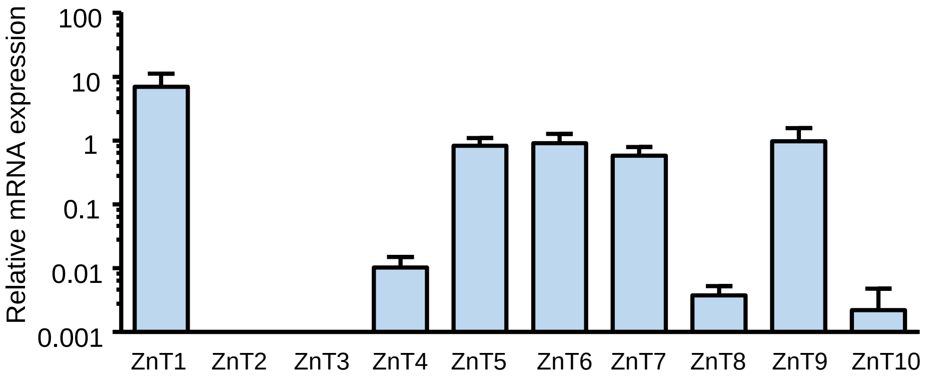

B

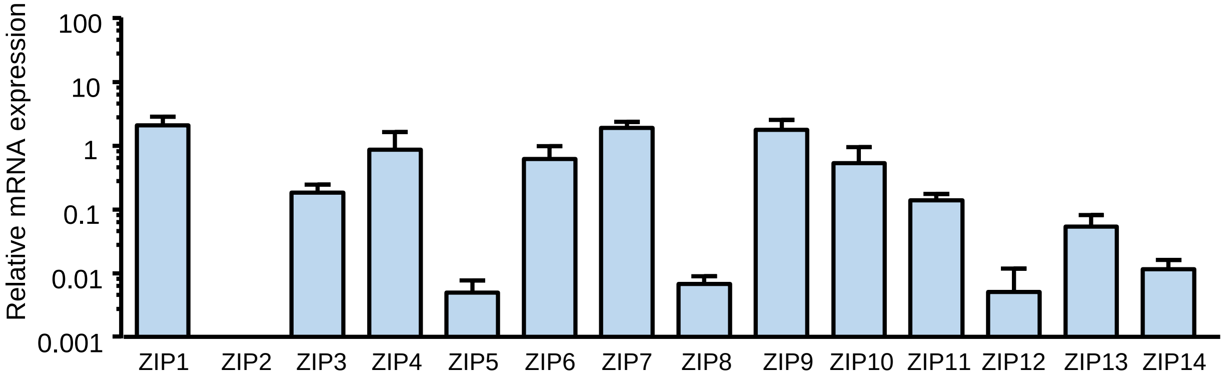

Supplementary Figure 3

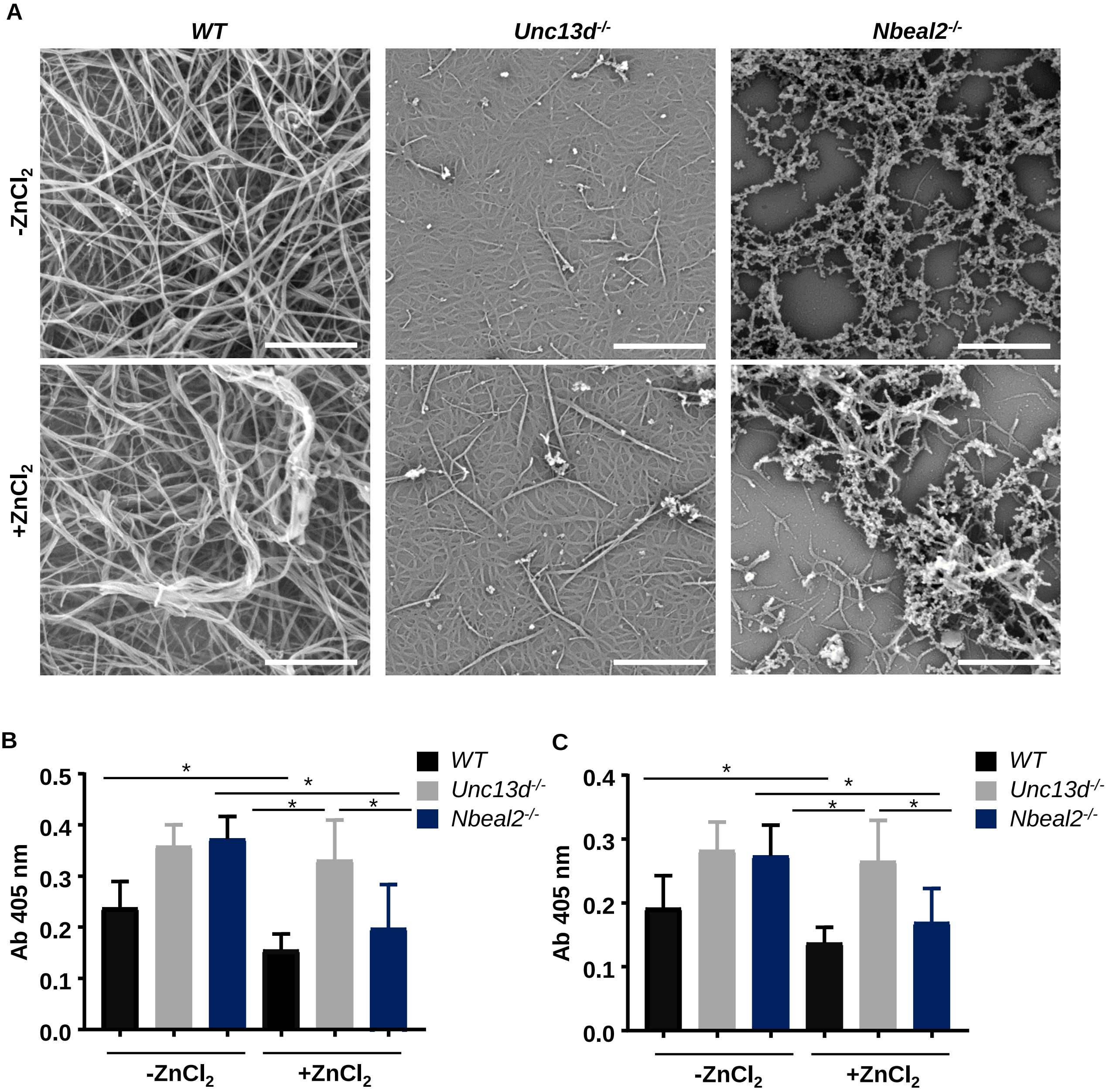

Supplementary Figure 4

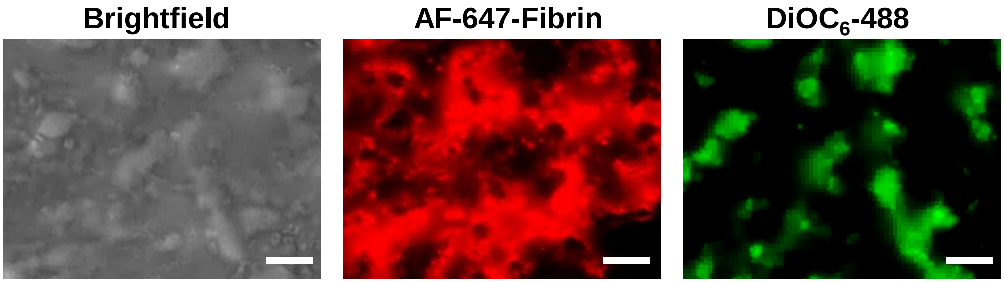

Supplementary Figure 5

ID	Age	Sex (1=M,2=W)	Bleeding score (ISTH-BAT)	Platelet count ($x10^9$/L) Reference 150-450	MPV (fL) Reference: 81-99	PFA-100 Col/EPI (sec) Reference: 84-160	PFA-100 Col/ADP (sec) Reference: 68-121	Agg-Col (AU) [1µg/mL] Reference: 76-95%	Agg-TRAP (AU) [20µmol/L] Reference: 68-97%	Agg-ADP (AU) [10µmol/L] Reference: 59-95%	ATP release (µM/10^{12} platelets) Reference: > 10.1	ADP amount (µM/10^{12} platelets) Reference: > 49.1
GF1	28	2	3	194	11.1	> 296	99	6	35	58	n.d.	n.d.

Supplementary Table 1

ID	Age	Sex (1=M,2=W)	Bleeding score (ISTH-BAT)	Platelet count ($x10^9/L$) Reference 150-450	MPV (fL) Reference: 81-99	PFA-100 Col/EPI (sec) Reference: 84-160	PFA-100 Col/ADP (sec) Reference: 68-121	Agg-Col (AU) [1µg/mL] Reference: 76-95%	Agg-TRAP (AU) [20µmol/L] Reference: 68-97%	Agg-ADP (AU) [10µmol/L] Reference: 59-95%	ATP release ($µM/10^{12}$ platelets) Reference: > 10.1	ADP amount ($µM/10^{12}$ platelets) Reference: > 49.1
418	6	1	2	274	10.6	162	108	38	58	46	n.d.	n.d.
419	7	1	4	300	10.6	115	n.d.	35	61	48	0.8	56.4
420	8	1	3	472	10.2	194	146	66	88	61	2.8	127.1
426	7	1	2	427	9.9	118	n.d.	7	62	23	4.1	80.1
428	9	1	3	281	10.1	120	n.d.	23	53	52	4.8	236.5
456	7	1	3	223	10.2	164	151	10	27	16	3.4	85.6
457	9	1	3	382	9.7	115	n.d.	37	63	35	n.d.	n.d.
458	14	2	3	281	11.7	n.d.	n.d.	n.d.	n.d.	n.d.	n.d.	n.d.
466	8	2	1	257	10.8	175	113	n.d.	n.d.	n.d.	n.d.	n.d.
467	12	2	3	284	10.6	146	n.d.	n.d.	n.d.	n.d.	n.d.	n.d.

Supplementary Table 2

Primer	Sequence	Amplicon Size
mRplp0 Forward	5’-GAAACTGCTGCCTCACATCCG-3’	146 bp
mRplp0 Reverse	5’-CTGGCACAGTGACCTCACACG-3’	
mZIP1 Forward	5’-ATTCCCGACGCGAGGC-3’	146 bp
mZIP1 Reverse	5’-TCCGATGCGACTGCTTCTG-3’	
mZIP2 Forward	5’-CAGATGGATGCAGCTACAGGTC-3’	82 bp
mZIP2 Reverse	5’-CTGCTCCCAAGAAGACACCTG-3’	
mZIP3 Forward	5’-GTCCCTCTGCAACACCTTCG-3’	147 bp
mZIP3 Reverse	5’-ACCATCATGAGCGTCTCCG-3’	
mZIP4 Forward	5’-GGCCTGTTAAATACGCTGGTG-3’	136 bp
mZIP4 Reverse	5’-TCTGGACTCCAGGACTGATTCTG-3’	
mZIP5 Forward	5’-TGGGAGATTGCCTGCACA-3’	147 bp
mZIP5 Reverse	5’-CCTGAAGCAGCATGGCAA-3’	
mZIP6 Forward	5’-CACGCCTCGTAAAGGCTTCTC-3’	134 bp
mZIP6 Reverse	5’-ATCTGTGGCCATTGCACCTT-3’	
mZIP7 Forward	5’-GGTTGCGGAAAAGGAGAGG-3’	137 bp
mZIP7 Reverse	5’-AAGTTGTGTGCCAAGTCAGCAG-3’	
mZIP8 Forward	5’-CGGACACATCCACTTCGACA-3’	129 bp
mZIP8 Reverse	5’-AGCGTGATCATCCAGGCAAT-3’	
mZIP9 Forward	5’-GGAGGGCAGAATGGATGACTT-3’	150 bp
mZIP9 Reverse	5’-ACAGAGAAGACCAGCACCCAG-3’	
mZIP10 Forward	5’-GAGCGGAGAGGAGATGCACA-3’	145 bp
mZIP10 Reverse	5’-GGCCATGGTCATGGTCTTCA-3’	
mZIP11 Forward	5’-GGGCACCCTACAGTATCCAGC-3’	150 bp
mZIP11 Reverse	5’-GCTTCCGTCTAAGATCCGCC-3’	
mZIP12 Forward	5’-GCCAGCCTCCAACAGAAATG-3’	142 bp
mZIP12 Reverse	5’-TGTTGTGGTCACTCCTGACTCC-3’	
mZIP13 Forward	5’-CTGCCACCTCTGCCACTG-3’	130 bp
mZIP13 Reverse	5’-GGGAACCTCCAGCCCTTTC-3’	
mZIP14 Forward	5’-CCTGGCTATTGGTGCCTCC-3’	119 bp
mZIP14 Reverse	5’-CAGCATTGAGCAGGATGACG-3’	
mZnT1 Forward	5’-AATTCCAACGGGCTGAAGG-3’	150 bp
mZnT1 Reverse	5’-TCCTCGCATATTCAGCTGCC-3’	
mZnT2 Forward	5’-CCCATCTGCACCTTCCTCTTC-3’	139 bp
mZnT2 Reverse	5’-CCACGGACAGCAGGAGATTT-3’	
mZnT3 Forward	5’-GGAGACCACCCGCCTAGTG-3’	120 bp
mZnT3 Reverse	5’-ATCCCCTCAAGCTTGGGCT-3’	

mZnT4 Forward	5’-ATGCACTCCATATGCTAACTGACC-3’	134 bp
mZnT4 Reverse	5’-ACACTGATCATGGCCGACAA-3’	
mZnT5 Forward	5’-ACCACTAAGGACCTTGCTGCTG-3’	149 bp
mZnT5 Reverse	5’-CGAAAAGCAGTAAGCAGATCACA-3’	
mZnT6 Forward	5’-TGGGAAAGTGCTCCTCCAGAC-3’	131 bp
mZnT6 Reverse	5’-GAGCCAAATCCAAGGGTCC-3’	
mZnT7 Forward	5’-GGCAAGATCTCAGGCTGGTTT-3’	136 bp
mZnT7 Reverse	5’-GATCAAGCCTAGGCAGTTGCTC-3’	
mZnT8 Forward	5’-AGTTGATGGCGTGATCTCCG-3’	130 bp
mZnT8 Reverse	5’-TTGAGCAATTCCTGTCCGC-3’	
mZnT9 Forward	5’-AGGCGCAGAACTCAAAGCTC-3’	150 bp
mZnT9 Reverse	5’-CACTGGACTTAAGGCAGAACTCG-3’	
mZnT10 Forward	5’-GGTGATTCCCTGAACACCGA-3’	139 bp
mZnT10 Reverse	5’-TAGCCGTGATGACCACAACC-3’	

Supplementary Table 3. List of primer sequences
